# High-Sensitive C-Reactive Protein Levels in a Group of Syrian University Male Students and Its Associations with Smoking, Physical Activity, Anthropometric Measurements, and Some Hematologic Inflammation Biomarkers

**DOI:** 10.1155/2017/7326527

**Published:** 2017-04-10

**Authors:** Wafika Zarzour, Nada Dehneh, Mazen Rajab

**Affiliations:** Biochemistry Department, Faculty of Pharmacy, Arab International University, Damascus, Syria

## Abstract

In Syria, health risk data on young males are limited. Hence, the aim of the present study was to evaluate cardiovascular disease (CVD) risk factors along with C-reactive protein levels measured by high-sensitive method (hsCRP) in a group of healthy males of university students (*n* = 101, 18–25 years old). Participants' anthropometric characteristics; alcohol drinking, smoking, and physical activity habits; parents medical history; and some inflammatory biomarkers were inspected for their associations with hsCRP.* Results*. Regarding hsCRP level, 19 participants were at average (1–3 mg/L) and 13 were at high (>3 mg/L) risk of CVD. Nonparametric statistical tests (*p* value < 0.05) revealed that hsCRP level was higher in participants who had high body mass index (BMI), had high BMI with high waist-to-hip ratio (WHR), or did not practice sport frequently. Unexpectedly, it did not vary between smokers and nonsmokers. In general, it correlated positively with anthropometric and erythrocyte sedimentation rate (ESR) measurements. Nevertheless, it negatively correlated with sports practicing in overall and nonsmoker groups and in participants whose parents were without medical history. Finally, when participants with high BMI were smokers, did not practice sport frequently, or had a parent with medical history, their hsCRP levels were higher than others who had the same circumstances but with low BMI.

## 1. Introduction

It seems that the inflammatory process plays an essential role in all phases of atherosclerosis development from initiation to clinical complications. This process begins in early childhood and can last throughout life [1].

Hence, anthropometric measurements, lifestyle, and inflammatory biomarkers levels' screening at early adulthood can provide prognostic information on health risks.

C-reactive protein levels measured by high-sensitive method (hsCRP) is an acute phase reactant protein, a marker of systemic inflammation, or possible participant in the inflammatory process [2]. It has been associated with risk factors for cardiovascular disease (CVD), particularly, with lifestyle factors such as smoking and physical activity [3], and it has been shown to be a predictor of cardiovascular events [4]. In addition, clinical studies had demonstrated that chronic inflammation is an independent risk factor for metabolic syndrome and CVD [5].

Nevertheless, few studies have reported associations between hsCRP and CVD risk factors among adolescents and young adults. More specifically, in Syria, health risk information on young people is limited and little is known about lifestyle factors and biomarkers related to CVD risk. Previously, we inquired into some of the CVD risk factors in young women in Syria (Dehneh et al., 2016).

In this study, we targeted young male adults aged between 18 and 25 years, who present 16.76% of men and 8.6% of the population in Syria [6].

In these young men, we investigated some of the CVD risk factors, specially, hsCRP. Also, we studied some of hsCRP correlations with anthropometric measurements, physical activity, smoking, parent medical history for CVD or diabetes, and some of the hematological inflammation biomarkers such as blood cells count (WBC), erythrocyte sedimentation rate (ESR), mean platelet volume (MPV), neutrophil to lymphocyte ratio (NLR), and red cells distribution width (RDW).

## 2. Materials and Methods

### 2.1. Subjects

During the period from 15 January to 30 April, 2016, male students in the Faculty of Pharmacy at Arab International University (AIU), Damascus, Syria, were informed about the aim of the study. Among 300 male students, one hundred and one (*n* = 101) healthy male students agreed to participate in the study providing written informed consent and fully answered the questionnaire of the study. A descriptive cross-sectional design was used and the following criteria were adopted: the volunteer's age should be between 18 and 25 years, the volunteer should have neither acute nor chronic medical history (such as diagnosed CVD, cerebrovascular disease, dyslipidemia, stable hypertension treated by drugs, chronic hepatic disease, and renal problems) and should not be taking any kind of medications during the previous two months. The retrieved data were confidential.

### 2.2. Anthropometric, Clinical, and Lifestyle Characteristics

In the presence of the research team, each participant filled in a questionnaire about his age, smoking habit, physical activity, medication, and personal and parental medical history.

Waist and hip circumferences and height were measured to the nearest 0.5 cm, and weight was measured with a lever balance to the nearest 100 g. BMI (kg/m^2^) was calculated as weight (kg) divided by the square of the standing height (m^2^). Waist-to-hip ratio (WHR) was calculated as waist circumference divided by hip circumference.

Alcohol drinking, current cigarette or hubble-bubble (water pipes, shisha, nargile, and hookah have similar structures in which the smoke passes through water, causing a bubbling sound) smoking, and physical activity status were assessed by frequency questionnaire.

Information about parent medical history was positive for 50 participants; they had at least the mother or the father affected with one of the following diseases: type 2 diabetes, CVD, dyslipidemia, and hypertension.

Waist circumference is an indicator of health risk associated with excess fat around the waist. A waist circumference greater than 102 cm (35 inches) in men is associated with health problems such as type 2 diabetes, heart disease, and high blood pressure [7–9]. Also, world health organization (WHO) standards state that abdominal obesity for males is defined as a WHR equal to or more than 0.90 or a BMI more than 30 kg/m^2^ [10].

Hence, results of waist circumference and WHR were classified by their risks of disease and cross-tabulated with BMI obesity classification. The numbers of participants with the median of hsCRP levels of each crossed class were reported.

The results of categorical variables of lifestyle such as alcohol drinking, sports practicing, and hubble-bubble smoking were grouped by the participants' habit frequencies (rarely, monthly, weekly, and daily). Sports were separated into sports that need sports club and others that do not. Also, cigarette smoking habit was expressed in terms of number of cigarettes smoked per day. The median of hsCRP levels and numbers of participants of each group were reported.

### 2.3. Laboratory Analyses

Laboratory analyses were performed at the medical laboratory of AIU. At 8-9 AM, venipuncture was performed for each participant by applying a latex rubber strap and using a 10 mL syringe. 4 mL of blood was immediately transferred to one tube for serum separation, 2.5 mL to a second tube containing the anticoagulant ethylene diamine tetraacetic tripotassium salt (K_3_EDTA) for complete blood counting, and 2 mL to a third tube containing sodium citrate as anticoagulant for ESR measurement.

ESR was measured by the Westergren method [11]. Complete blood count was performed using an automated hematology analyzer ABX MICRO 60 [12] and hsCRP by nephelometric technology on the BN ProSpec System, a fully automated bench top analyzer. The manufacturer claims that lower limit of detection and limit of quantification (LOQ) of hsCRP levels are 0.18 mg/L [13]. In this study, the minimum observation of hsCRP (0.11 mg/L) was 39% of LOQ and nonparametric statistical methods were used; therefore, levels lower than the LOQ (5 observations) were included in the investigation.

Hematological participants' results were grouped by low, normal, and high levels of hematological age-specific reference ranges established by Department of Pathology, School of Medicine, Virginia Commonwealth University (UCV) [14].

hsCRP results of participants were grouped by low, average, and high risk levels of American Heart Association (AHA) and US Centers for Disease Control and Prevention recommendations [15]. The median of hsCRP levels and the number of participants of each group were reported.

### 2.4. Statistical Analysis

To summarize the results of continuous variables like anthropometric and hematological characteristics of participants, the mean and median were calculated to evaluate the central tendency of variables. To describe the dispersion of those variables, the standard deviation (SD), minimum, and maximum were calculated; first and third quartiles values (Q1–Q3) were reported as the boundaries of the middle half of a set of data. The normal distribution for each variable was assessed using Shapiro Wilk and Kolmogorov–Smirnov tests. Statistical significance was defined at *p* < 0.05.

To recognize hsCRP levels and other measurements patterns among participants, principle component analysis could not be applied because the validity of all possible correlation matrices and samples sizes' (number of participants into different groups) tests showed too little correlation between variables (calculated Bartlett's test for homogeneity of variance* p* values were more than 0.05). Also, simple or multiple linear regression models could not be applied because the residuals of the fitted linear regression models were not normally distributed; hence, the assumption of normal distribution of prediction errors is not valid. In addition, multiple linear regression models may need larger number of participants.

Hence, to distinguish the effect of BMI, WHR, MPV, smoking, type of smoking, sport, and parent medical history, for each factor and by using appropriate inclusion criteria, the participants were divided into two opposite groups. To note any differences between groups, Wilcoxon rank sum test (nonparametric) for independent samples was performed.

To study the associations of hsCRP levels with other variables without the assumption of binormality between two variables or any particular distribution, a nonparametric correlation coefficient was needed. The widely used Kendall's tau-b coefficient is easier to understand, interpret, and explain than other correlation coefficients.

Kendall's tau-b correlation coefficients between hsCRP levels and other variables were calculated and hypothesis testing for Kendall's tau-b using normal approximation was used to evaluate the importance of correlation coefficient values. Statistical significance was defined at *p* < 0.05.

Finally, to investigate the effect of participants' BMI along with smoking, sports practicing, and the presence of medical history in one of the participants' parents on hsCRP levels, each group of participants was separated, according to BMI levels of participants, into two groups. Wilcoxon rank sum test of significance difference (*p* value) was applied between different pairs of groups and only* p* values < 0.05 were retained. Excel software 2007 (Microsoft) was used for the statistics calculations.

## 3. Results

### 3.1. Anthropometric Characteristics and Results of Laboratory Analyses

Tables 1 and 2 show anthropometric measurements and results of laboratory analyses, respectively. Approximately, all variables had normal distribution except for ESR and hsCRP results, which were skewed to low levels.

### 3.2. Distribution of High-Sensitive CRP Results 

#### 3.2.1. Distribution of hsCRP Levels in Groups of BMI along with Waist Circumference and WHR

Waist circumference, WHR, BMI, and risk levels along with the distribution of median of hsCRP levels were presented in Table 3.

Our results demonstrated that, among 101 participants, overweight and obesity were present in 46 participants; waist circumference levels more than 102 cm were present in 10 participants only. These participants had high median of hsCRP levels.

WHR's results showed that 36 participants had WHR ≥ 0.90. In addition to them, 7 participants had, approximately, BMI > 30 kg/m^2^ and WHR < 0.90. Overall, 43 participants had abdominal obesity and average levels of hsCRP (>1 mg/L) were present in 11 of them.

Wilcoxon rank sum test, where it was applicable, did not show significance difference (*p* value < 0.05) between any group of participants and the overall group.

#### 3.2.2. Distribution of hsCRP Levels along with Habits of Drinking, Smoking, Sports Practicing, and Parent Medical History

Table 4 shows heavy cigarette smokers (5 participants) had hsCRP levels more than 1 mg/L.

Levels of hsCRP in participants shown in Table 5 were grouped by parent(s) medical history. Occurrence of hypertension, dyslipidemia, CVD, and type 2 diabetes was studied since these diseases are risk factors for CVD. 50 participants had at least one parent affected by one of the studied disease.

#### 3.2.3. Distribution of hsCRP Levels along with Hematological Biomarkers Levels

Table 6 shows hematological participant's results grouped by low, normal, and high levels of hematological age-specific reference ranges established by UCV [14].

Only hsCRP levels were grouped according to the AHA recommendations for hsCRP testing and relative risk of CVD to low, average, or high categories which correspond to approximate hsCRP concentrations < 1.0, 1.0 to 3.0, and >3.0 mg/L, respectively [15].

These reference values were used due to the absence of their equivalents, locally.

27, 29, and 16 participants showed low levels of MCV, MCH, and MCHC, respectively. Elevated values of MPV were found in 36 participants and 14 participants had RBCs more than 5.78*∗*10^12^ cells/L. As for hsCRP results, 19 participants were at average risk (hsCRP within 1–3 mg/L) and 13 were at high risk (hsCRP < 3 mg/L) for CVD. Wilcoxon rank sum test, where it was applicable, did not show significance difference (*p* value < 0.05) between any group of participants and the overall group.

### 3.3. Correlation between Anthropometric Measurements, Physical Activity, Hematological Biomarkers, and hsCRP Levels

In order to study the effect of BMI, WHR, MPV, smoking, type of smoking, sport, and participant's parent medical history, on hsCRP levels, participants were grouped using appropriate criteria.

Table 7 shows hsCRP levels were significantly higher in high BMI, high BMI accompanied with high WHR, and no sports practicing groups compared to their counterparts.

Unexpectedly, hsCRP levels were not significantly different between smokers and nonsmokers.

Also, significant positive correlations for hsCRP levels along with anthropometric measurements were present.

Nevertheless, significant (*p* value < 0.05) negative correlations for hsCRP levels along with sports practicing in overall, nonsmokers, and parent without medical history groups, as well as significant negative correlations for hsCRP levels along with MPV in low BMI and low BMI accompanied with high WHR groups, were noticed.

Unexpectedly, hsCRP levels did not significantly correlate with any inflammatory markers or anthropometric measurements in groups of participants who had low BMI with low WHR, had parents without medical history, or were hubble-bubble smokers. In addition, the remaining groups, except for low BMI with high WHR, high BMI with low WHR, and frequent sports practicing groups, were marked by the significant positive correlation between hsCRP and ESR levels.

In particular, positive correlation between hsCRP and ESR was significant (*p* value < 0.025) in normal values group of MPV and of lesser degree of significance (0.025 ≤* p* value < 0.05) in the opposite group (high MPV).

It is worth noting that levels of MPV were significantly higher in the group of cigarette smokers (*n* = 30) compared with hubble-bubble smokers group (*n* = 23),* p* value = 0.007 (results not shown in the table).

### 3.4. hsCRP Levels according to BMI with Smoking, Physical Activity, and Parent Medical History Status

**Figure 1:**
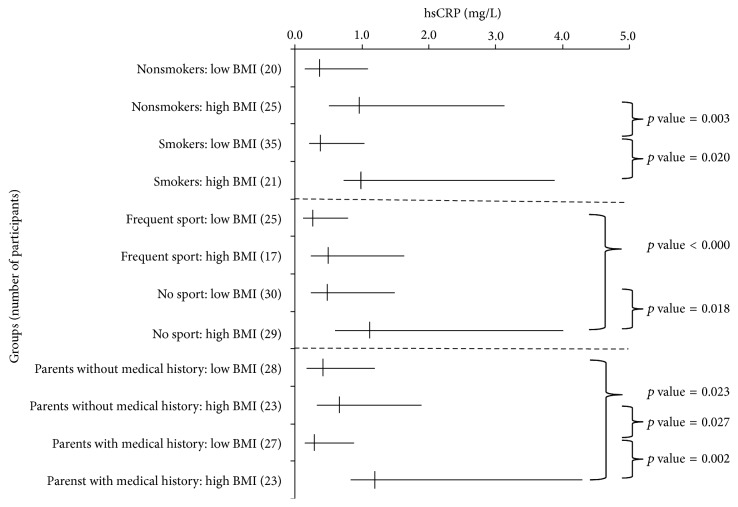
To investigate the effect of the participant's BMI along with smoking, sports practicing, and the presence of medical history in one of the participant's parents on hsCRP levels, each group of participants was separated, according to their BMI levels, into two groups. Vertical lines denote the median; horizontal lines represent participant's group enclosed in interquartile range. Wilcoxon rank sum test of significance difference (*p* value) was applied between different pairs of groups and only *p* values < 0.05 were shown.

As shown in Figure 1, there were no significant differences in hsCRP levels between participants with high BMI and those who had low BMI levels when both high BMI and low BMI participants were nonsmokers, practiced sport frequently, or had parents without medical history. On the contrary, when participants with high BMI were smokers, did not practice sport frequently, or had a parent with medical history, their hsCRP levels were significantly higher than others with the same circumstances but with low BMI.

In addition, hsCRP levels were significantly lower in participants with low BMI who practiced sport frequently or had parents without medical history compared to those with high BMI who did not practice sport frequently or had a parent with medical history, respectively. Even the hsCRP levels in nonsmokers were, apparently, low as shown in Figure 1 but were not significantly lower with low BMI compared to their smoking counterparts with high BMI.

On the other hand, hsCRP levels were significantly higher in participants with high BMI who were nonsmokers or had parents without medical history compared to those with low BMI who were smokers or had a parent with medical history, respectively. Contrarily, hsCRP levels were not significantly higher in participants with high BMI who practiced sport frequently compared to their non-sport practicing counterparts with low BMI.

It is worth noting that there were no significant differences in hsCRP levels between low BMI groups. The same is also noted for hsCRP levels in participants with high BMI.

## 4. Discussion

The present study was conducted on a group of apparently healthy young men; it showed that 19 participants were at average risk (hsCRP within 1–3 mg/L) and 13 were at high risk (hsCRP >3 mg/L) for CVD. These results are comparable with the findings of several studies [4, 16].

In addition, several studies have pointed to a positive correlation between hsCRP and intensity of the atheromatous process and the risk of cardiovascular events occurring as atheromatous complications [1] and data from prospective, epidemiologic studies revealed a significant association between CRP and future coronary heart disease risk in apparently healthy subjects [17].

In the present investigation, hsCRP levels were negatively correlated with sport practicing and positively correlated with BMI and waist and hip circumferences each. Furthermore, the study of physical activity revealed that the levels of sport practicing in the studied group were low; only 24 of the participants practiced sport daily, which could explain the occurrence of obesity in the studied group. Nevertheless, the levels of hsCRP in frequently practicing sport group were significantly lower than the opposite group where hsCRP concentrations correlated significantly with BMI, weight, and waist and hip circumferences. Therefore, in the absence of sportive activity, obesity indices could be positively associated with CRP levels.

On the contrary, the negative correlation between hsCRP concentrations and sport practicing was obvious in overall, nonsmokers, and participants with parents without medical history groups.

Our results were similar to Kuo H-K's findings who revealed that in adults CRP concentration levels were inversely related to cardiorespiratory fitness [18]. In other studies, no relation was found between hsCRP concentration and the physical activity [1]. Meanwhile, the results of Stewart et al. study had supported the use of combined aerobic/resistance training as a modality to reduce the risk of CVD development as defined by a decrease in serum CRP concentration in healthy humans [19]. Possibly, various exercise regimens may influence inflammatory markers differently, and there is still a lack of knowledge about the target exercise level leading to optimum anti-inflammatory efficiency [20].

It is accepted that adipose tissue is a major source of inflammatory cytokines such as interleukin-6 (IL-6) and tumor necrosis factor-*α* (TNF-*α*). Consequently, CRP is made by the liver in response to these inflammatory cytokines. In overall group, 10 participants had waist circumference > 102 cm with overweight or obesity and 36 were abdominally obese (WHR > 0.9) and our results showed significant positive correlations for hsCRP along with BMI and waist and hip circumferences

These results were partially in agreement with Lapice study that showed that, in case of healthy nonobese people, hsCRP was associated with abdominal adiposity independently of age and BMI (Lapice et al., 2009).

However, in the present study, since hsCRP concentrations were significantly higher in high BMI (>25 kg/m^2^) and high BMI combined with high WHR groups than their counterparts, respectively, generalized obesity effect on hsCRP levels is more pronounced than the abdominal obesity alone. These findings are in concordance with many published studies [21–24]. In addition, a strong positive association has been found between measures of obesity, such as waist circumference and BMI, with CRP [17, 25].

Our results showed no significant difference in hsCRP levels between the participants group with clean parent medical history and group with parent medical history of diabetes, CVD, hypertension, and dyslipidemia. Yet, the effect of parent medical history on hsCRP levels was previously studied. Pannacciulli found that, in a group of women free from risk factors for atherothrombosis, subjects with a family history of type 2 diabetes have higher hsCRP plasma levels than age- and BMI-matched controls with no family history [26]. So, the limited number of participants in our study could have biased our result.

Higher levels of hsCRP were observed in 5 participants who were classified as heavy smokers, but this group was too small to suggest a dose-dependent relation between smoking and hsCRP levels. No significant effect of cigarette or hubble-bubble smoking on CRP levels was noted in our study.

These results contradict the classical idea that cigarette smoking is a major risk factor in the development of several diseases with an inflammatory component, including CVD and chronic obstructive pulmonary disease [27], although a relation between tobacco smoke exposure status or active smoking and CRP levels was observed by several studies [28–30].

Our results displayed significant correlations between hsCRP levels and inflammatory markers such as WBC and ESR in the overall group.

It is well known that WBC count and hsCRP concentration are basic indicators of systemic inflammation. Elevated levels of WBC count are associated with increased risk for morbidity and mortality in middle-aged and old populations [31]. A number of prospective epidemiological studies had demonstrated that hsCRP, as well as WBC count, can independently predict vascular risk in both apparently healthy men and women and patients with clinical signs of CVD [32].

Our results showed that correlation between hsCRP and WBC was independent of abdominal obesity. However, the positive and significant correlation between hsCRP and WBC, existent in the overall group, was also present in the groups of participants who practiced sports frequently, had low WHR, or were smokers. The influence of smoking was reported, also, by Asthana who noted that hsCRP levels and WBC counts are higher among current smokers compared to never smokers [33].

No correlation between CRP concentration and NLR was found in our study group of healthy young adults, except in the group of high BMI and that of high MPV which is a group of proinflammatory milieu. On the contrary, recent evidence indicated that the ratio of subtypes of blood cells have a significant prognostic value for CVD. So, NLR could be an important measure of systemic inflammation as it is cost-effective, readily available, and could be calculated easily [34]. In addition, it has been shown that elevated NLR is associated with increased concentration of various proinflammatory cytokines [35].

ESR, a nonspecific test, constitutes one of the oldest laboratory methods. An accelerated ESR may be indicative of inflammation or the presence of a tumor [36]. In the absence of confounding conditions, ESR appears to be a strong short- and long-term predictor of coronary heart disease mortality in apparently healthy, middle-aged men [37].

The correlation between hsCRP and ESR was present in our studied sample. A similar finding was shown in a group of healthy males and females, younger than 40 years, in a study conducted by Osei-Bimpong [38].

As indicated by our results, hsCRP and ESR correlation is independent of obesity, visceral obesity, and smoking status, although it is shown that the combination of high BMI with high WHR influenced positively the occurrence of this correlation. It was reported that obesity was associated with inflammation process activation [25].

Very recently, RDW was found to be a strong, independent predictor of morbidity and mortality in large heart failure populations [39]. RDW is an automatically measured index of the heterogeneity of the erythrocytes. A strong, graded association between RDW and two widely used plasma inflammatory biomarkers, such as hsCRP and ESR, was found in a large cohort of unselected adult outpatients [40]. However, in our study, no association was found between CRP levels and those of RDW; the youth of participants may be the cause of this absence.

In addition, platelets are known to have a major effect on the formation of atherosclerotic plaques and therefore play an essential role in the pathogenesis of atherothrombosis. Platelet size and activity are correlated, and MPV was found to be increased before acute myocardial infarction [41].

The importance of MPV has been emphasized as an inflammation marker in some chronic inflammatory disorders, such as inflammatory intestinal diseases and rheumatoid arthritis [42].

In our study, no correlation between CRP and MPV was found in the sample, but we noted that in some participants this correlation was negative especially in participants groups of low BMI and low BMI accompanied with high WHR. On the contrary, this correlation was positive in high BMI, high BMI accompanied with low WHR, and high BMI accompanied with high WHR groups. Generalized obesity could have an activating effect on platelets. Coban, in 2005, had reported that MPV was positively correlated with BMI in obese group, and in 2007, he revealed that there was a positive correlation between weight loss and reduction in MPV [43, 44].

Smoking cession had been accompanied by MPV decreasing [45, 46]. It is worth noting that, as previously mentioned (Section 3.3), the significant difference in MPV levels between cigarette smokers and hubble-bubble smokers needs further investigation. However, it does not eliminate the bad effects of hubble-bubble smoking.

The correlations between CRP and anthropometric measures (waist and hip circumferences, weight, BMI, and WHR) were noted in the overall group and in several groups except nonsmokers, normal MPV, parent without medical history, and frequently practicing sport groups, which suggests that these conditions could also affect the CRP's levels.

However, the absence of these correlations in low BMI and high BMI groups demonstrated the strong association between CRP levels and BMI status, which is in concordance with Delongui findings [24].

In numerous literatures, hsCRP levels were studied along with only one condition at a time. In this study, when two conditions were combined, high levels of BMI were present when hsCRP levels' difference between two opposite groups was significantly increased. This association was clear when high BMI is combined with nonfrequent sport practicing, the presence of a parent with medical history, or smoking conditions.

The combination between conditions accompanied with high hsCRP levels (high BMI with no sport practicing and high BMI with parent medical history) was correlated with significantly higher levels of hsCRP when compared to opposite groups, respectively. It is surprising that this rise was statistically absent when high BMI combined with smoking was compared to low BMI combined with no smoking group.

It seems that the participant's parents medical history, as well as smoking status, had an influence, partially and independently of his BMI, on hsCRP levels. On the contrary, BMI is usually associated with sport practicing. Hence, the independence between these two characteristics was less pronounced.

Unfortunately, due to the descriptive design of the study, we could not study the evolution of hsCRP level, and its effect as a risk factor for CVD in our group. As the limited number of participants, their young age, and their situation as students could affect the results, hence this article needs larger investigations to emphasize its results.

## 5. Conclusion

In this study, conducted on a group of healthy young male adults, hsCRP levels were closely distributed as it is shown in the literature. While hsCRP levels were significantly higher in participants with high BMI and high BMI combined with high WHR groups, cigarette or hubble-bubble smoking were not associated with increased hsCRP concentrations. Although sports practicing's association with hsCRP levels is still undefined, the association between frequent sports practicing and low levels of hsCRP was noted in the studied group.

Even though the association of medical parent history with hsCRP levels was reported in this work, it was not completely independent of the effect of BMI.

To view the role of the inflammatory markers in the prediction of future CVD, our study showed a positive correlation between hsCRP levels and ESR in this group of young age.

Finally, a general advice for young men in Syria is to consider a healthy lifestyle rich in physical activity and free of smoking.

## Figures and Tables

**Table 1:** Anthropometric characteristics of the participants (*n* = 101); the results are presented as mean ± standard deviation (SD), median, first and third quartiles (Q1–Q3), minimum, and maximum. Damascus, Syria, April 2016.

Characteristic, unit	Mean ± SD	Median (Q1–Q3)	Minimum	Maximum
Age, year	21.63 ± 2.10^#^	22 (20–23)	18	25
Height, cm	176.24 ± 7.12^#*∗*^	176 (171–180)	159	203
Weight, kg	79.44 ± 13.08^†#^	78 (67.4–87.7)	54.7	118.0
BMI, kg/cm^2^	25.60 ± 4.39^#^	24.62 (22.76–27.97)	16.99	38.53
Waist, cm	90.58 ± 10.29^#*∗*^	89 (84–98)	71	120
Hip, cm	103.91 ± 8.60^#*∗*^	104 (97–109)	85	133
WHR ratio	0.87 ± 0.05^#*∗*^	0.87 (0.83–0.91)	0.75	0.98

^†^After the elimination of 2 outliers, which were out of the range of 2.5th and 97.5th percentiles, the normal distribution was proved using Shapiro-Wilk test (*p* < 0.05). Only mean ± SD were recalculated.

^#^Normal distribution was proved by Kolmogorov-Smirnov test (*p* < 0.05).

^*∗*^Normal distribution was proved by Shapiro-Wilk test (*p* < 0.05).

**Table 2:** Hematological characteristics of the participants (*n* = 101); the results are presented as mean ± standard deviation (SD), median, first and third quartiles (Q1–Q3), minimum, and maximum. Damascus, Syria, April 2016.

Characteristic, unit	Mean ± SD	Median (Q1–Q3)	Minimum	Maximum
WBC, 10^9^/L	7.06 ± 1.71^#^	6.7 (5.9–8.0)	4.1	12.4
Red blood cell count, 10^12^/L	5.44 ± 0.38^#^	5.38 (5.17–5.63)	4.31	6.69
Hemoglobin, g/dL	14.97 ± 1.19^#^	15.1 (14.4–15.8)	10.8	17.7
Hematocrit, %	45.01 ± 2.83^†#^	45.2 (43.3–47.1)	33.8	51.4
Mean red cell volume, fL	83.22 ± 6.33^#^	84 (81–87)	60	95
Mean red cell hemoglobin, pg	27.67 ± 2.50^*ʃ*^	27.9 (26.8–29.0)	17.8	32.4
Mean red cell hemoglobin conc., g/dL	33.17 ± 1.05^#*∗*^	33.1 (32.7–33.9)	29.8	35.8
Platelet, 10^9^/L	240.13 ± 45.93^#*∗*^	238 (203–274)	149	389
MPV, fL	8.77 ± 0.74^#*∗*^	8.7 (8.3–9.2)	7.4	11.0
RDW, %	12.63 ± 0.69^*ʃ*^	12.6 (12.1–12.9)	11.4	19.2
Monocytes, %	4.6 ± 1.1^#^	4.5 (3.8–5.3)	2.5	7.3
Neutrophils, %	63.16 ± 8.68^#*∗*^	63.3 (57.6–68.3)	42.3	86.5
Lymphocytes, %	32.24 ± 8.12^#*∗*^	32 (27.3–36.9)	11	52
NLR ratio	2.14 ± 0.91^*ʃ*^	1.99 (1.56–2.48)	0.81	7.86
ESR, mm in 1 hour	3.47 ± 4.25^§^	2 (1–4)	1	33
hsCRP, mg/L	1.52 ± 3.10^§^	0.51 (0.24–1.31)	0.11	19.80

^*ʃ*^After the elimination of 2 outliers, which were out of the range of 2.5th and 97.5th percentiles, the normal distribution was proved using Kolmogorov-Smirnov test (*p* < 0.05). Only mean ± SD were recalculated.

^†^After the elimination of 2 outliers, which were out of the range of 2.5th and 97.5th percentiles, the normal distribution was proved using Shapiro-Wilk test (*p* < 0.05). Only mean ± SD were recalculated.

^#^Normal distribution was proved by Kolmogorov-Smirnov test (*p* < 0.05).

^*∗*^Normal distribution was proved by Shapiro-Wilk test (*p* < 0.05).

^§^No normal distribution, mean ± SD was cited without any further interpretation.

**Table 3:** Participant's anthropometric measurements grouped by BMI, waist circumference [8] and WHR [10]. Median of hsCRP levels (mg/L) and participant numbers (*n*) of each group were shown (*n* = 101). Disease risk^*∗*^ of BMI and waist circumference were reported. Damascus, Syria, April 2016.

BMI (kg/m^2^), class	Median of hsCRP levels mg/L (*n*) (Disease risk)				
		Waist circumference		WHR	
		≤102 cm	>102 cm	<0.90	≥0.90
	Totals	0.47 (91)	1.77 (10)	0.47 (65)	0.57 (36)
<18.5, Underweight	0.12 (2)	0.12 (2) (Non)	— (0) (^§^)	0.12 (2)	— (0)
18.5–24.9, Normal	0.38 (53)	0.38 (53) (Non)	— (0) (^§^)	0.37 (38)	0.38 (15)
25.0–29.9, Overweight	0.80 (28)	0.59 (26) (Increased)	5.17 (2) (High)	0.80 (18)	0.74 (10)
30.0–34.9, Obese I	1.16 (16)	0.93 (10) (High)	1.35 (6) (Very high)	0.95 (7)	1.34 (9)
35.0–39.9, Obese II	2.38 (2)	— (0) (Very high)	2.38 (2) (Very high)	— (0)	2.38 (2)
40 or greater, Extremely Obese III	— (0)	— (0) (Extremely high)	— (0) (Extremely high)	— (0)	— (0)

^*∗*^Disease risk for type 2 diabetes, hypertension, and CVD relative to normal weight and waist circumference.

— empty group

^§^Increased waist circumference also can be a marker for increased risk, even in persons of normal weight.

Note: Wilcoxon rank sum test, where it was applicable, did not show significance difference (*p*-value < 0.05) between any group of participants and the overall group.

**Table 4:** Participants grouped by alcohol drinking, smoking, or sport habits characteristics. Median of hsCRP levels (mg/L) and participants numbers (*n*) of each group were shown (*n* = 101). Damascus, Syria, April 2016.

Habit	Median of hsCRP levels mg/L (*n*)					
	Yes	No	Habit frequencies			
			Rarely	Monthly	Weekly	Daily
Alcohol drinking	0.41 (5)	0.52 (96)	0.41 (5)	— (0)	— (0)	— (0)
Sport	0.48 (70)	0.68 (31)	0.74 (23)	1.04 (5)	0.47 (19)	0.38 (23)
Sport that need						
No club	0.59 (43)		0.81 (22)	1.11 (3)	0.29 (15)	0.40 (3)
Club	0.38 (27)		0.12 (1)	0.59 (2)	0.92 (4)	0.38 (20)
Smoking	0.43 (56)	0.55 (45)				
Smoking by tobacco type						
Hubble-bubble smoking^§^	0.47 (23)		0.38 (1)	— (0)	0.38 (10)	0.63 (12)
Number of cigarettes per day (*x*)			0 < *x* ≤ 5	5 < *x* ≤ 10	10 < *x* ≤ 20	*x* > 20
Cigarettes	0.37 (30)		0.40 (7)	0.26 (7)	0.33 (11)	1.34 (5)
Both	0.65 (3)					

^§^Water pipes, shisha, nargile, and hookah have similar structures in which the smoke passes through water, causing a bubbling sound.

**Table 5:** Participants grouped by the occurrence of diseases in their parents. Median of hsCRP levels (mg/L) and participants numbers (*n*) of each group were shown (*n* = 101). Damascus, Syria, April 2016.

Occurrence of diseases in participant's parents	Median of hsCRP levels mg/L (*n*)				
	Hypertension	Dyslipidemia	CVD^*∗*^	Diabetes type 2	One disease at least
None	0.51 (68)	0.50 (75)	0.51 (86)	0.52 (83)	0.52 (51)
Mother only	0.55 (13)	0.55 (11)	1.77 (1)	0.27 (8)	0.45 (22)
Father only	0.51 (13)	0.37 (13)	0.35 (13)	0.35 (9)	0.48 (24)
Both	0.45 (7)	1.64 (2)	2.37 (1)	1.36 (1)	0.95 (14)
One of the parents at least	0.51 (33)	0.53 (26)	1.19 (15)	0.36 (18)	0.48 (50)

^*∗*^Cardiovascular diseases.

**Table 6:** Participants' hematological measurements grouped by low, normal, or high levels of hematological reference values of VCU^*∗*^ reference ranges. Median of hsCRP levels (mg/L) and participants numbers  (*n*)  of each group were shown  (*n* = 101). Damascus, Syria, April 2016.

Characteristic, unit	VCU^*∗*^			
	[Normal range]	Low	Normal	High
		Median of hsCRP levels mg/L (*n*)		
WBC, 10^9^/L	[3.7–9.7]	— (0)	0.52 (93)	0.40 (8)
RBC, 10^12^/L	[4.54–5.78]	3.76 (1)	0.52 (86)	0.48 (14)
Hemoglobin, g/dL	[13.3–17.2]	1.38 (6)	0.50 (94)	0.51 (1)
Hematocrit, %	[38.9–50.9]	2.07 (4)	0.50 (95)	0.41 (2)
MCV, fL	[81.2–94.0]	0.45 (27)	0.55 (73)	0.15 (1)
MCH, pg	[27.1–32.5]	0.52 (29)	0.50 (72)	— (0)
MCHC, g/dL	[32.5–36.7]	0.55 (16)	0.50 (85)	— (0)
Platelet, 10^9^/L	[179–373]	0.35 (5)	0.51 (95)	1.04 (1)
MPV, fL	[6.1–8.9]	— (0)	0.58 (65)	0.43 (36)
RDW, %	[11.5–14.1]	1.80 (2)	0.50 (94)	0.58 (5)
Monocytes, %	[3.3–9.2]	0.52 (12)	0.51 (89)	— (0)
Neutrophils, %	[42.9–78.4]	0.24 (1)	0.52 (93)	0.41 (7)
Lymphocytes, %	[14.1–45.8]	0.41 (1)	0.56 (94)	0.18 (6)
ESR (mm in 1 hour)	[0–15]	— (0)	0.50 (99)	3.42 (2)

	AHA^*∗∗*^			
	[Average risk]	Low risk	Average risk	High risk
		Median of hs-CRP levels mg/L (*n*)		

hsCRP, mg/L	[1–3]	0.30 (69)	1.36 (19)	4.55 (13)

^*∗*^Hematological age-specific reference ranges established by Department of Pathology, School of Medicine, Virginia Commonwealth University [17].

—: empty group.

^*∗∗*^The American Heart Association and US Centers for Disease Control and Prevention recommendations for hsCRP testing and CVD risks [13].

Note: Wilcoxon rank sum test, where it was applicable, did not show significance difference (*p* value < 0.05) between any group of participants and the overall group.

**Table 7:** Using appropriate inclusion criteria, the participants were divided into two opposite groups. To check the difference significance of hsCRP levels (mg/L) between them, Wilcoxon rank sum test was applied. To reveal the possible associations, Kendall's tau-b correlation coefficients between hsCRP and other variables were used^*∗*^.

Group name	Group criteria	*n* ^†^	hsCRP median (Q1–Q3)^‡^ mg/L	Wilcoxon rank-sum test *p* value^*∗∗*^	hsCRP associated with the following: Variable name (Kendall's tau-b correlation coefficient) ^a,b,c^
Overall	No condition	101	0.51 (0.24–1.31)		Sport (−0.19)^b^, BMI (0.19)^b^, waist (0.22)^b^, hip (0.20)^b^, WBCs (0.17)^b^, ESR (0.25)^b^.

Low BMI	BMI < 25 kg/m^2^	55	0.37 (0.16–0.66)		MPV (−0.28)^b^, NLR (0.19)^c^, ESR (0.23)^c^.
High BMI	BMI ≥ 25 kg/m^2^	46	0.97 (0.32–2.39)	0.002	MPV (0.31)^b^, ESR (0.32)^b^.

Low WHR	WHR < 0.9	65	0.47 (0.22–1.11)		Waist (0.19)^c^, WBCs (0.18)^c^, ESR (0.21)^c^.
High WHR	WHR ≥ 0.9	36	0.57 (0.26–1.84)	0.387	BMI (0.24)^c^, waist (0.32)^b^, hip (0.31)^b^, ESR (0.32)^b^.

Low BMI with low WHR	BMI < 25 and WHR < 0.9	40	0.35 (0.15–0.67)		No correlation with inflammatory markers or anthropometric measures.
High BMI with high WHR	BMI ≥ 25 and WHR ≥ 0.9	21	0.98 (0.26–3.08)	0.017	MPV (0.37)^b^, ESR (0.37)^c^.

Low BMI with high WHR	BMI < 25 and WHR ≥ 0.9	15	0.38 (0.28–0.59)		MPV (−0.52)^b^.
High BMI with low WHR	BMI ≥ 25 and WHR < 0.9	25	0.94 (0.41–2.18)	0.094	MPV (0.32)^c^.

Normal MPV	6.1 ≤ MPV ≤ 8.9 fL	65	0.58 (0.25–1.19)		ESR (0.24)^b^.
High MPV	MPV > 8.9 fL	36	0.43 (0.20–1.45)	0.563	Weight (0.37)^b^, BMI (0.50)^a^, waist (0.44)^a^, hip (0.45)^a^, NLR (0.27)^b^, ESR (0.28)^c^.

Nonsmokers	Nonsmokers	45	0.55 (0.26–1.36)		Sport (−0.27)^b^, ESR (0.27)^b^.
Smokers	Smokers	56	0.43 (0.20–1.15)	0.280	BMI (0.19)^c^, waist (0.24)^b^, hip (0.22)^b^, WBCs (0.20)^c^, ESR (0.27)^b^.

Cigarette smokers	Cigarettes only	30	0.37 (0.21–1.28)		Weight (0.28)^c^, BMI (0.30)^b^, waist (0.35)^b^, hip (0.34)^b^, ESR (0.29)^c^.
Hubble-bubble smokers	Hubble-bubble smoking only	23	0.47 (0.18–0.90)	0.740	No correlation with inflammatory markers or anthropometric measures.

Frequent sport	Weekly or more frequent	42	0.38 (0.23–0.58)		WBCs (0.33)^b^.
No sport	Monthly or less frequent	59	0.68 (0.32–1.98)	0.011	Weight (0.21)^b^, BMI (0.23)^b^, waist (0.28)^b^, hip (0.27)^b^, ESR (0.28)^b^.

Parents without medical history	No parents medical history for participant	51	0.52 (0.24–1.08)		No correlation with inflammatory markers or anthropometric measures.
Parents with medical history	At least one parent had medical history	50	0.48 (0.23–1.56)	0.461	Sport (−0.21)^c^, weight (0.29)^b^, BMI (0.28)^b^, waist (0.36)^a^, hip (0.36)^a^, ESR (0.39)^a^.

^*∗*^Only correlation coefficients that have *p* value < 0.05 were reported.

^†^Number of participants in the group.

^‡^Q1 and Q3 are first and third quartiles of the group, respectively.

^*∗∗*^Except for the group that contains all participants, Wilcoxon rank sum test of significance difference (*p* value) was applied between every two successive groups.

^a^ *p* value < 0.001.

^b^0.001 ≤ *p* value < 0.025.

^c^0.025 ≤ *p* value < 0.05.
